# Evaluation of Project P.A.T.H.S. (Secondary 1 Program) by the Program Implementers: Findings Based on the Full Implementation Phase

**DOI:** 10.1100/tsw.2008.70

**Published:** 2008-04-29

**Authors:** Daniel T. L. Shek, Hing Keung Ma

**Affiliations:** ^1^ Centre for Quality of Life, Hong Kong Institute of Asia-Pacific Studies, The Chinese University of Hong Kong, Hong Kong; ^2^ Department of Sociology, East China Normal University, China; ^3^ Kiang Wu Nursing College of Macau, Macau; ^4^ Department of Education Studies, Hong Kong Baptist University, Hong Kong

**Keywords:** child health, human development, adolescence, youth development, Chinese

## Abstract

A total of 207 schools (N = 35,735 students) participated in the Secondary 1 Program of Project P.A.T.H.S. in the full implementation phase (2006/07 school year). After completion of the Tier 1 Program, 1,250 instructors completed a subjective outcome evaluation form (Form B) to assess their views of the program, instructors, and perceived effectiveness of the program. Utilizing the consolidated reports submitted to the funding body, the research team aggregated the consolidated data to form an overall profile of the perceptions of the program participants. Results showed that high proportions of the respondents had positive perceptions of the program and the instructors, and roughly four-fifths of the respondents regarded the program as helpful to the program participants and the workers. These findings complement the subjective outcome evaluation findings based on the perspective of the program participants.

## INTRODUCTION

In different human services, such as education, social work, psychology, medicine, and allied health professions, subjective outcome evaluation has commonly been used to evaluate program effectiveness. Although there are many criticisms of this approach, there are several arguments supporting its use in program evaluation. First, it is economical and few financial resources are needed. Second, it is simple and not sophisticated in terms of research designs. Third, unlike evaluation that utilizes experimental paradigms, there is no need to use sophisticated statistical analyses. Fourth, there are research findings that show that the client satisfaction approach could yield useful findings if valid and reliable measures were used. Finally, there are research findings that show that clients' perceived benefits of the program were moderately associated with objective outcome evaluation findings[1].

According to utilization-focused evaluation, it is important to understand the views of the stakeholders[2]. As program implementers are stakeholders of the program, it is logically important to examine the experiences of workers who conduct the intervention or implement the program. This practice is particularly important for programs that are implemented by workers who are not directly involved in the design of the program. In the context of positive youth development or adolescent prevention programs, the programs are usually developed by academics and experienced workers in the field (e.g., school-based drug prevention programs). The developed programs are then implemented by front-line workers, such as teachers and social workers. Under such contexts, front-line workers may have strong resistance and doubts in implementing programs because they have little involvement in the design process. Furthermore, organizational constraints, such as increased workload, may also adversely affect staff morale, which, in turn, lowers the motivation of the workers to implement the program in an authentic manner. As such, it is important to understand the views of program implementers regarding the program implementation process.

There are several other arguments supporting the collection of subjective evaluation data from the program implementers. First, as program implementers are usually more experienced than the clients, it can be argued that their views may be more accurate than those of the clients. With more professional skills, training, and experience, workers implementing the program may be in a better position to assess the effectiveness of the programs. Second, the inclusion of subjective outcome evaluation based on the worker's perspective can give the workers a sense of fairness and respect, which would enhance the morale of the workers. When the workers are invited to express their views and their voices are heard, they would feel more respected, thus not regarding themselves as the victims of consumerism. Third, if the researchers can build up a systematic profile of the experiences of the workers and disseminate the related findings, the related research findings can demystify the rumors and distorted news about the program (e.g., the program does not work; one must substantially change the program before successful implementation), which are commonly found in adolescent prevention and positive youth development programs. Politically speaking, subjective outcome evaluation based on the perspective of the program implementers can help to provide a transparent and accurate picture on the implementation quality, which can further engage the program implementers in a meaningful manner. Finally, based on the principle of triangulation, collection of subjective outcome evaluation data from different data sources (e.g., program participants and program implementers) definitely can help to increase the credibility of the evaluation data collected. It can be argued that if the students perceive that the program works well, but the workers do not have a similar perception, the effectiveness of the program is questionable[3,4].

To promote holistic development among adolescents in Hong Kong, the Hong Kong Jockey Club Charities Trust approved HK$400 million to launch a project entitled "P.A.T.H.S. to Adulthood: A Jockey Club Youth Enhancement Scheme"[5]. There are two tiers of programs (Tier 1 and Tier 2) in the P.A.T.H.S. Project (P.A.T.H.S. = Positive Adolescent Training through Holistic Social Programmes). The Tier 1 Program is a universal positive youth development program where students in Secondary 1 to 3 will participate, normally with 20 h of training in the school year at each grade involving 40 teaching units that are developed with reference to 15 positive youth development constructs[6,7]. There are two implementation phases in this project: Experimental Implementation Phase and Full Implementation Phase. For the Experimental Implementation Phase, 52 secondary schools participated in the project with the objectives of accumulating experience in program implementation and familiarizing front-line workers with the program design and philosophy. Following the Experimental Implementation Phase, 207 schools joined the Secondary 1 Program in the Full Implementation Phase.

Based on the principle of triangulation, evaluation data based on different strategies and sources have been collected to understand the program effectiveness and effect.

1. **Objective Outcome Evaluation**: Both one group pre-/post-test design[8] and randomized group trial were adopted to evaluate the effectiveness of the program[9].
2. **Subjective Outcome Evaluation**: Both students and program implementers responded to the subjective outcome evaluation forms (Form A and Form B, respectively) after completion of the program. The existing quantitative[3,4] and qualitative findings[10,11] showed that different stakeholders perceived the program to be beneficial to the participants. The subjective outcome evaluation findings were also intimately related to objective outcome evaluation findings[1].

3. **Process Evaluation**: Systematic observations were carried out by trained observers in randomly selected schools to understand the program implementation details. The findings generally revealed that the implementation quality and program adherence was high[12,13,14].
4. **Interim Evaluation**: To understand the process of implementation, interim evaluation was conducted by randomly selecting half of the participating schools. The findings are generally positive and encouraging, and they provide additional process evaluation data based on the school observations[15,16,17].
5. **Qualitative Evaluation (Focus Groups Based on Students)**: Focus groups involving students based on schools randomly selected from the participating schools were carried out. Results showed that the comments of program participants were generally positive, although there were also some suggestions for improvement[18,19].
6. **Qualitative Evaluation (Focus Groups Based on Program Implementers)**: Focus groups involving instructors based on schools randomly selected from the participating schools were carried out. Results showed that the comments of the workers were generally positive, although there were also some suggestions for improvement.
7. **Qualitative Evaluation (In-Depth Interviews with Program Implementers)**: Prolonged in-depth interviews with two teachers were carried out.
8. **Qualitative Evaluation (Case Study Based on Focus Groups)**: A case study based on seven schools participating in the Secondary 1 Program of the Full Implementation Phase was conducted. In these cases, the Tier 1 Program had been successfully incorporated into school formal curriculum[20,21].
9. **Qualitative Evaluation (Student Logs)**: Four students were invited to reflect on their experiences after joining the classes and application of things learned to real life.
10. **Qualitative Evaluation (Student Products)**: Students' weekly diaries were collected after completion of the program[22]. Students' drawings were also collected to reflect the experiences of the program participants.

Although the existing subjective outcome evaluation findings based on program implementers are encouraging[3], such findings are based on 52 schools in the Experimental Implementation Phase only. To replicate such findings and to give a broader view of the issue, subjective outcome evaluation data based on program implementers were collected from 207 schools participating in the Full Implementation Phase (Secondary 1 Level). As the Project P.A.T.H.S. was financially supported by the Hong Kong Jockey Club Charities Trust, each participating school was required to submit an evaluation report with the consolidated subjective outcome evaluation profile of the students as well as the workers to the funding body. Based on the submitted information, an overall profile of the views of the program implementers was reconstructed in an anonymous manner.

## METHODS

### Participants and Procedures

There were 207 secondary schools joining the Secondary 1 Program in the Full Implementation Phase (2006/07). The mean number of students per school was 172.63 (range: 17–280 students), with an average of 4.66 classes per school (range: 1–8 classes). Among them, 112 schools adopted the full program (i.e., 20-h program involving 40 units), while the remaining adopted the core program (i.e., 10-h program involving 20 units). The mean number of sessions used to implement the program was 23.55 (range: 2–50 sessions). While 101 (48.79%) schools incorporated the program in the formal curriculum (e.g., Liberal Studies, Life Education), 106 schools (51.21%) used other modes (e.g., form master's periods and other combinations) to implement the program. The mean numbers of social workers and teachers implementing the program per school were 2.13 (range: 0–9) and 5.47 (range: 0–14), respectively.

After the Tier 1 Program was completed, the workers were invited to respond to a subjective outcome evaluation questionnaire. A total of 1,250 social workers and teachers responded to the Subjective Outcome Evaluation Form (Form B) developed by the research team. The data collection was normally carried out after the completion of the program. To facilitate the program evaluation, the research team developed an evaluation manual with standardized instructions for collecting the subjective outcome evaluation data[23]. In addition, adequate training was provided to the workers during the 20-h training workshops on how to collect and analyze the data collected by Form B.

## Instruments

The Subjective Outcome Evaluation Form (Form B) was designed by Daniel Shek and Andrew Siu[23]. Broadly speaking, there are several parts in this evaluation form as follows:

- Program implementers' perceptions of the program, such as program objectives, design, classroom atmosphere, interaction among the students, and the students' participation during class (10 items).
- Program implementers' perceptions of their own practice, including their understanding of the course, teaching skills, professional attitude, involvement, and interaction with the students (10 items).
- Workers' perceptions of the effectiveness of the program, such as promotion of different psychosocial competencies, resilience, and overall personal development of the students (16 items).
- The extent to which the worker would recommend the program to other students with similar needs (1 item).
- The extent to which the worker would teach similar programs in future (1 item).
- The extent of which the program has enhanced the worker's professional growth (1 item).
- Things that the worker obtained from the program (open-ended question).
- Things that the worker appreciated most (open-ended question).
- Difficulties encountered (open-ended question).
- Areas that require improvement (open-ended question).

The workers collecting the data were requested to input the data into an EXCEL file developed by the research team, which would automatically compute the frequencies and percentages associated with the different ratings for an item. When the schools submitted the reports, they were also requested to submit the soft copy of the consolidated data sheets. After receiving the consolidated data by the funding body, the data were aggregated to "reconstruct" the overall profile based on the subjective outcome evaluation data.

## RESULTS

Reliability analysis with the schools as the unit of analyses showed that the Form B was internally consistent: 10 items related to the program (alpha = 0.93, mean interitem correlation = 0.55), 10 items related to the instructor (alpha = 0.87, mean interitem correlation = 0.41), 16 items related to the benefits (alpha = 0.97, mean interitem correlation = 0.66), and 39 items based on the whole Form B (alpha = 0.97, mean interitem correlation = 0.47).

The quantitative findings based on the closed-ended questions are presented in this paper. There are several observations that can be highlighted from the findings. First, the workers generally had positive perceptions of the program (Table 1), including the objectives of the teaching units (94.5%), systematic design of the teaching activities (80.8%), and active involvement of the students (84.5%). Second, a high proportion of the workers had positive evaluation of their performance (Table 2). For example, 96% of the workers had positive evaluation of their performance, 98.8% of the workers expressed that they were concerned about the students, 96.9% believed that they had very good professional attitude as an instructor. Third, as shown in Table 3, many workers perceived that the program promoted the development of students, including their bonding (91.0%), resilience (85.5%), social competence (93.3%), emotional competence (91.3%), moral competence (89.9%), self-understanding (94.3%), and overall development (93.9%). Fourth, 88.8% of the workers would recommend the program to students with similar needs. Fifth, 84.3% of the workers expressed that they would teach similar courses again in the future. Finally, roughly four-fifths of the respondents indicated that the program had enhanced their professional growth (Table 4).

## DISCUSSION

The purpose of this study was to evaluate the Tier 1 Program of Project P.A.T.H.S. via the subjective outcome evaluation approach based on the perspective of the program implementers. Generally speaking, the quantitative findings showed that a high proportion of the workers had positive perceptions of the program and themselves; roughly four-fifths of the respondents regarded the program as helpful to the program participants. The findings basically replicated those findings reported previously based on the perspective of the program implementers in the Experimental Implementation Phase[3]. In fact, an examination of the percentages of responses to different items revealed that the figures were very similar across different studies. Furthermore, the findings are generally consistent with those findings based on the program participants[4].

In the context of human services, there is a growing emphasis on the importance of understanding the views and experiences of the workers who conduct the intervention. For example, Winefield and Barlow[24] argued that monitoring staff perception was important because “staff have valuable first-hand experience of how, when, and how well programs work” (p. 898). With specific reference to school-based prevention programs, Peterson and Esbensen[25] pointed out that “because personnel, consciously or unconsciously, influence the effectiveness of prevention program lessons, it is important to assess their perceptions when evaluating a specific program to provide insight into the context in which the program operates” (p. 219). In view of the limited research studies documenting the perceptions of workers in positive youth development and adolescent prevention programs in the Chinese literature, the present study can be regarded as a useful contribution.

Although utilization of the subjective outcome evaluation or client satisfaction approach in evaluation has a long history in human services, there are arguments against the use of subjective outcome evaluation[26]. Nevertheless, there are several features in this study that may be used to counterargue against such criticisms. First, a very big sample was used in this study, with 1,250 workers in 207 participating schools. Such a big sample size substantially enhances the generalizability of the research findings and their credibility. Second, different aspects of subjective outcome, including views on the program, worker, and perceived effectiveness were covered in the study. The present findings also showed that the Form B rating items were reliable with reference to the sections and the whole scale. According to Royse[27], the lack of standardized assessment tools for conducting client satisfaction survey introduces biases for the client satisfaction approach. As such, he recommended the use of assessment tool with known reliability and validity that would “eliminate many of the problems found in hastily designed questionnaires” (p. 265). Third, as the findings reported in this paper were “reconstructed” based on the reports submitted by the participating schools anonymously, the possibility that the workers reported in an overcooperative manner was not high. Fourth, previous research findings based on the study have shown that subjective outcome evaluation findings actually converged with objective outcome evaluation findings[1]. Finally, from a triangulation point of view, the present findings converged with those reported previously. The findings basically suggest that subjective outcome evaluation based on either program participants or implementers in different student cohorts are rather consistent.

**TABLE 1**
**Summary of the Views of the Program Implementers About the Program**

| | 1 | | 2 | | 3 | | 4 | | 5 | | 6 | | Participants with Positive Responses (Option 4–6) | |
|---|---|---|---|---|---|---|---|---|---|---|---|---|---|---|
| | Strongly Disagree | | Disagree | | Slightly Disagree | | Slightly Agree | | Agree | | Strongly Agree | | | |
| | N | % | N | % | N | % | N | % | N | % | N | % | N | % |
| The objectives of the curriculum are very clear. (N = 1,250) | 2 | 0.16 | 11 | 0.88 | 56 | 4.48 | 274 | 21.92 | 799 | 63.92 | 108 | 8.64 | 1,181 | 94.48 |
| The design of the curriculum is very good. (N = 1,247) | 7 | 0.56 | 67 | 5.37 | 165 | 13.23 | 490 | 39.29 | 488 | 39.13 | 30 | 2.41 | 1,008 | 80.83 |
| The activities were carefully planned. (N = 1,248) | 3 | 0.24 | 39 | 3.13 | 118 | 9.46 | 445 | 35.66 | 593 | 47.52 | 50 | 4.01 | 1,088 | 87.18 |
| The classroom atmosphere was very pleasant. (N = 1,246) | 2 | 0.16 | 33 | 2.65 | 138 | 11.08 | 480 | 38.52 | 523 | 41.97 | 70 | 5.62 | 1,073 | 86.12 |
| There was much peer interaction among the students. (N = 1,246) | 3 | 0.24 | 39 | 3.13 | 167 | 13.40 | 486 | 39.00 | 492 | 39.49 | 59 | 4.74 | 1,037 | 83.23 |
| Students participated actively during lessons (including discussions, sharing, games, etc.). (N = 1,248) | 1 | 0.08 | 29 | 2.32 | 164 | 13.14 | 465 | 37.26 | 513 | 41.11 | 76 | 6.09 | 1,054 | 84.46 |
| The program has a strong and sound theoretical support. (N = 1,242) | 3 | 0.24 | 28 | 2.25 | 136 | 10.95 | 487 | 39.21 | 507 | 40.82 | 81 | 6.52 | 1,075 | 86.55 |
| The teaching experience I encountered enhanced my interest in the course. (N = 1,244) | 12 | 0.96 | 68 | 5.47 | 182 | 14.63 | 489 | 39.31 | 448 | 36.01 | 45 | 3.62 | 982 | 78.94 |
| Overall speaking, I have very positive evaluation of the program. (N = 1,248) | 14 | 1.12 | 75 | 6.01 | 207 | 16.59 | 484 | 38.78 | 429 | 34.38 | 39 | 3.13 | 952 | 76.28 |
| On the whole, students like this curriculum very much. (N = 1,246) | 11 | 0.88 | 55 | 4.41 | 204 | 16.37 | 514 | 41.25 | 428 | 34.35 | 34 | 2.73 | 976 | 78.33 |

**TABLE 2**
**Summary of the Views of the Program Implementers About Themselves**

| | 1 | | 2 | | 3 | | 4 | | 5 | | 6 | | Participants with Positive Responses (Option 4–6) | |
|---|---|---|---|---|---|---|---|---|---|---|---|---|---|---|
| | Strongly Disagree | | Disagree | | Slightly Disagree | | Slightly Agree | | Agree | | Strongly Agree | | | |
| | N | % | N | % | N | % | N | % | N | % | N | % | N | % |
| I have a good mastery of the curriculum. (N = 1,237) | 1 | 0.08 | 21 | 1.70 | 156 | 12.61 | 525 | 42.44 | 502 | 40.58 | 32 | 2.59 | 1,059 | 85.61 |
| I prepared well for the lessons. (N = 1,233) | 1 | 0.08 | 17 | 1.38 | 154 | 12.49 | 468 | 37.96 | 531 | 43.07 | 62 | 5.03 | 1,061 | 86.05 |
| My teaching skills were good. (N = 1,230) | 0 | 0.00 | 15 | 1.22 | 130 | 10.57 | 532 | 43.25 | 518 | 42.11 | 35 | 2.85 | 1,085 | 88.21 |
| I have good professional attitudes. (N = 1,233) | 0 | 0.00 | 5 | 0.41 | 33 | 2.68 | 312 | 25.30 | 776 | 62.94 | 107 | 8.68 | 1,195 | 96.92 |
| I was very involved. (N = 1,237) | 1 | 0.08 | 6 | 0.49 | 57 | 4.61 | 362 | 29.26 | 684 | 55.30 | 127 | 10.27 | 1,173 | 94.83 |
| I gained a lot during the course of instruction. (N = 1,234) | 5 | 0.41 | 31 | 2.51 | 171 | 13.86 | 530 | 42.95 | 438 | 35.49 | 59 | 4.78 | 1,027 | 83.23 |
| I cared for the students. (N = 1,237) | 0 | 0.00 | 4 | 0.32 | 11 | 0.89 | 211 | 17.06 | 816 | 65.97 | 195 | 15.76 | 1,222 | 98.79 |
| I was ready to offer help to students when needed. (N = 1,236) | 0 | 0.00 | 2 | 0.16 | 9 | 0.73 | 157 | 12.70 | 797 | 64.48 | 271 | 21.93 | 1,225 | 99.11 |
| I had much interaction with the students. (N = 1,234) | 0 | 0.00 | 13 | 1.05 | 86 | 6.97 | 465 | 37.68 | 592 | 47.97 | 78 | 6.32 | 1,135 | 91.98 |
| Overall speaking, I have very positive evaluation of myself as an instructor. (N = 1,236) | 3 | 0.24 | 11 | 0.89 | 35 | 2.83 | 342 | 27.67 | 776 | 62.78 | 69 | 5.58 | 1,187 | 96.04 |

Although the present observations can be interpreted as reflecting the effectiveness of the program, there are several possible alternative explanations. The first alternative explanation is “beauty on the beholder side” hypothesis. As the workers are the stakeholders and they are personally involved in implementing the program, they tend to look at the program effect and their own performance in a more favorable light. In particular, unfavorable evaluation would pose a threat to the professional self and self-esteem of the workers. However, as the workers were professional social workers and teachers, such biases should be minimal. The second alternative explanation is the “survival” hypothesis, which maintains that positive subjective outcome evaluation findings occurred as a result of the workers’ anxiety that the program would be cut if the evaluation findings were not positive. This possibility can be partially dismissed because the funding body has never linked funding with program success and there is no league table in the evaluation findings. The final alternative interpretation is that the workers may consciously respond in a “nice” manner to help the researchers to illustrate positive program effect. However, this alternative explanation could be partially dismissed because negative ratings were recorded

**TABLE 3**
**Perceived Effectiveness of the Program by the Program Implementers**

| The extent to which the Tier 1 Program (i.e., the program in which all students have joined ) has helped your students | 1 Unhelpful | | 2 Not Very Helpful | | 3 Slightly Helpful | | 4 Helpful | | 5 Very Helpful | | Participants with Positive Responses (Option 3–5) | |
|---|---|---|---|---|---|---|---|---|---|---|---|---|
| | N | % | N | % | N | % | N | % | N | % | N | % |
| It has strengthened students' bonding with teachers, classmates, and their families. (N = 1,247) | 3 | 0.24 | 109 | 8.74 | 667 | 53.49 | 428 | 34.32 | 40 | 3.21 | 1,135 | 91.02 |
| It has strengthened students' resilience in adverse conditions. (N = 1,244) | 8 | 0.64 | 172 | 13.83 | 679 | 54.58 | 356 | 28.62 | 29 | 2.33 | 1,064 | 85.53 |
| It has enhanced students' social competence. (N = 1,246) | 3 | 0.24 | 80 | 6.42 | 525 | 42.13 | 549 | 44.06 | 89 | 7.14 | 1,163 | 93.34 |
| It has improved students' ability in handling and expressing emotions. (N = 1,246) | 4 | 0.32 | 104 | 8.35 | 607 | 48.72 | 474 | 38.04 | 57 | 4.57 | 1,138 | 91.33 |
| It has enhanced students' cognitive competence. (N = 1,245) | 7 | 0.56 | 197 | 15.82 | 615 | 49.40 | 377 | 30.28 | 49 | 3.94 | 1,041 | 83.61 |
| Students' ability to resist harmful influences has been improved. (N = 1,245) | 6 | 0.48 | 213 | 17.11 | 647 | 51.97 | 340 | 27.31 | 39 | 3.13 | 1,026 | 82.41 |
| It has strengthened students' ability to distinguish between the good and the bad. (N = 1,247) | 4 | 0.32 | 122 | 9.78 | 610 | 48.92 | 456 | 36.57 | 55 | 4.41 | 1,121 | 89.90 |
| It has increased students' competence in making sensible and wise choices. (N = 1,246) | 4 | 0.32 | 176 | 14.13 | 619 | 49.68 | 401 | 32.18 | 46 | 3.69 | 1,066 | 85.55 |
| It has helped students to have life reflections. (N = 1,245) | 17 | 1.37 | 198 | 15.90 | 580 | 46.59 | 397 | 31.89 | 53 | 4.26 | 1,030 | 82.73 |
| It has reinforced students' self-confidence. (N = 1,247) | 7 | 0.56 | 204 | 16.36 | 609 | 48.84 | 377 | 30.23 | 50 | 4.01 | 1,036 | 83.08 |
| It has increased students' self-awareness. (N = 1,244) | 2 | 0.16 | 69 | 5.55 | 536 | 43.09 | 561 | 45.10 | 76 | 6.11 | 1,173 | 94.29 |
| It has helped students to face the future with a positive attitude. (N = 1,246) | 7 | 0.56 | 189 | 15.17 | 636 | 51.04 | 376 | 30.18 | 38 | 3.05 | 1,050 | 84.27 |
| It has helped students to cultivate compassion and care about others. (N = 1,243) | 9 | 0.72 | 180 | 14.48 | 639 | 51.41 | 380 | 30.57 | 35 | 2.82 | 1,054 | 84.79 |
| It has encouraged students to care about the community. (N = 1,243) | 15 | 1.21 | 254 | 20.43 | 658 | 52.94 | 278 | 22.37 | 38 | 3.06 | 974 | 78.36 |
| It has promoted students' sense of responsibility in serving the society. (N = 1,247) | 12 | 0.96 | 278 | 22.29 | 638 | 51.16 | 290 | 23.26 | 29 | 2.33 | 957 | 76.74 |
| It has enriched the overall development of the students. (N = 1,246) | 6 | 0.48 | 70 | 5.62 | 599 | 48.07 | 498 | 39.97 | 73 | 5.86 | 1,170 | 93.90 |

(e.g., whether the workers would teach similar courses again) and the workers responded in an anonymous manner.

Despite these limitations, the present findings suggest that the Tier 1 Program of the Secondary 1 Program and its implementation were perceived in a positive manner by the program implementers. In conjunction with other evaluation findings, the present study suggests that the Tier 1 Program of Project P.A.T.H.S. was beneficial to the holistic development of the program participants. With reference to the gradual decline of parental control in the early adolescent years in Chinese adolescents in Hong Kong,

**TABLE 4**
**Other Aspects of Subjective Outcome Evaluation Based on the Views of the Workers**

If you have a student/client whose needs and conditions are similar to those of your students who have joined the program, will you suggest him/her to participate in this program? (N = 1,233)

| 1 | | 2 | | 3 | | 4 | | Participants with Positive Responses (Option 3–4) | |
|---|---|---|---|---|---|---|---|---|---|
| Definitely Will Not Suggest | | Will Not Suggest | | Will Suggest | | Definitely Will Suggest | | | |
| N | % | N | % | N | % | N | % | N | % |
| 13 | 1.05 | 125 | 10.14 | 969 | 78.59 | 126 | 10.22 | 1095 | 88.81 |

If there is a chance, will you teach similar programs again in the future? (N = 1,223)

| 1 | | 2 | | 3 | | 4 | | Participants with Positive Responses (Option 3–4) | |
|---|---|---|---|---|---|---|---|---|---|
| Definitely Will Not Teach | | Will Not Teach | | Will Teach | | Definitely Will Teach | | | |
| N | % | N | % | N | % | N | % | N | % |
| 23 | 1.88 | 169 | 13.82 | 905 | 74.00 | 126 | 10.30 | 1031 | 84.30 |

Do you think the implementation of the program has helped you in your professional growth (e.g., enhancement of your skills)? (N = 1,231)

| 1 | | 2 | | 3 | | 4 | | 5 | | Participants with Positive Responses (Option 3–5) | |
|---|---|---|---|---|---|---|---|---|---|---|---|
| Unhelpful | | Not Very Helpful | | Slightly Helpful | | Helpful | | Very Helpful | | | |
| N | % | N | % | N | % | N | % | N | % | N | % |
| 29 | 2.36 | 192 | 15.60 | 603 | 48.98 | 355 | 28.84 | 52 | 4.22 | 1010 | 82.05 |

positive youth development programs such as Project P.A.T.H.S. are important initiatives to promote their psychosocial competencies[28].

## ACKNOWLEDGMENTS

The preparation for this paper and Project P.A.T.H.S. were financially supported by the Hong Kong Jockey Club Charities Trust.
